# Manmade Electromagnetic Fields and Oxidative Stress—Biological Effects and Consequences for Health

**DOI:** 10.3390/ijms22073772

**Published:** 2021-04-06

**Authors:** David Schuermann, Meike Mevissen

**Affiliations:** 1 Department of Biomedicine, University of Basel, Mattenstrasse 28, CH-4058 Basel, Switzerland; 2 Veterinary Pharmacology and Toxicology, Vetsuisse Faculty, University of Bern, Laenggassstrasse 124, CH-3012 Bern, Switzerland

**Keywords:** oxidative stress, ROS, electromagnetic field, extremely low frequency, radiofrequency, environment and public health, environmental exposure, animal study, cultured cells

## Abstract

Concomitant with the ever-expanding use of electrical appliances and mobile communication systems, public and occupational exposure to electromagnetic fields (EMF) in the extremely-low-frequency and radiofrequency range has become a widely debated environmental risk factor for health. Radiofrequency (RF) EMF and extremely-low-frequency (ELF) MF have been classified as possibly carcinogenic to humans (Group 2B) by the International Agency for Research on Cancer (IARC). The production of reactive oxygen species (ROS), potentially leading to cellular or systemic oxidative stress, was frequently found to be influenced by EMF exposure in animals and cells. In this review, we summarize key experimental findings on oxidative stress related to EMF exposure from animal and cell studies of the last decade. The observations are discussed in the context of molecular mechanisms and functionalities relevant to health such as neurological function, genome stability, immune response, and reproduction. Most animal and many cell studies showed increased oxidative stress caused by RF-EMF and ELF-MF. In order to estimate the risk for human health by manmade exposure, experimental studies in humans and epidemiological studies need to be considered as well.

## 1. Introduction

Reactive oxygen species (ROS), as well as related reactive nitrogen species (RNS), are involved in many biological processes; nonetheless, they pose a hazard to the biological material and physiology of cells [1,2,3]. Protective mechanisms, such as antioxidants and antioxidative enzymes, maintain physiological concentrations of ROS in cells, while external and internal stimuli affect the amount of ROS by altering the activity of involved ROS-forming and -degrading enzymes [4]. For example, an increased energy requirement during physical activity leads to a temporary state of oxidative stress, and many environmental risk factors such as ionizing radiation in ultraviolet (UV) light or the radioactivity spectrum partly act via the formation of ROS. Pathophysiological levels of ROS interfere with many vital cellular processes and functions, such as inflammation, cell proliferation and differentiation, wound healing, neuronal activity, reproduction, and behavior by altering biochemical and signaling processes or even resulting in oxidative damage to DNA, RNA, and proteins or to the peroxidation of fatty acids [5,6]. If this unfavorable state persists over a long period or occurs repeatedly, it can lead to changes in the biological material, as well as the genetic and epigenetic information, and it can lead to health-related malfunctions. Accordingly, altered ROS levels and changes in biomarkers of oxidative stress as cause or consequence have been observed in many diseases, such as cancer, diabetes, congenital malformations, or neurodegenerative syndromes [1,3].

The influence of electromagnetic fields (EMF), as a manmade environmental factor with increasing importance, on ROS formation, triggering oxidative stress, has been repeatedly discussed. Corresponding hypotheses and experimental findings have been summarized and discussed in previous reviews on this topic [7,8,9,10,11,12,13,14,15,16]. Although there is consistent evidence for EMF-induced ROS formation in experimental studies, a complete picture and a scientific consensus have not yet emerged with regard to epidemiological association and possible negative and long-term consequences for health.

In this review, recently published relevant animal and cell studies were identified and evaluated with the aim toward providing an updated assessment of a causality between oxidative stress and exposure to magnetic and electromagnetic fields and their possible effects on health. The focus was put on environmentally and technologically relevant frequency ranges: extremely-low-frequency magnetic fields (ELF-MF) typical for 50/60 Hz alternating current (AC) power lines and radiofrequency electromagnetic fields (RF-EMF) in the frequency range from 800 MHz to 2.5 GHz as used for current mobile communication systems. This primarily involved experimental studies in animals and cultured and/or primary cells published in the peer-reviewed literature from 2010–2020 (Supplementary Materials, Tables S1–S4). These studies provided data about the influence of exposure on the formation of ROS, markers of oxidative stress, and changes in protective mechanisms that counteract oxidative stress.

Some studies are purely descriptive or contain mechanistic aspects that specifically track and investigate correlations and influenced processes. In animal experimentations, the balance of ROS and the antioxidant counterparts in the whole organism can be studied. In addition, functional changes, which are mostly based on a permanent imbalance and are, therefore, important for health, can be evaluated in animal studies. In addition to investigations on biomarkers of oxidative stress, molecular, morphological, or functional changes, such as induced DNA damage, impaired learning and memory, organ abnormalities, and decreased sperm count or motility are more conclusive for estimating possible adverse health effects. Therefore, studies showing functional changes are considered particularly important for estimating the impact of EMF on human health.

In the following chapters, we summarize important findings from animal and cell studies on oxidative stress and EMF exposure by organ system and related cell types, and we assess their relevance for human health. Furthermore, general aspects are included, which are independent of cell type and/or organ/tissue but need to be considered for such an assessment.

For this narrative review, a subset of animal and cell studies published in the last 10 years in English language that were considered relevant for the research question were assessed and included, in order to provide an overview of the current research. The included studies were extracted from databases available at BERENIS (https://www.bafu.admin.ch/bafu/en/home/topics/electrosmog/newsletter-of-the-swiss-expert-group-on-electromagnetic-fields-a/beratende-expertengruppe-nis-berenis.html, accessed on 10 June 2020), EMF Portal (https://www.emf-portal.org/en, accessed on 25 June 2020), and PubMed (https://pubmed.ncbi.nlm.nih.gov, accessed on 30 June 2020).

## 2. Background Information on Oxidative Stress

The chemical processes of oxidation and reduction are the basis for all biochemical reactions that make biological actions and life possible. The relatively reactive molecular oxygen in our atmosphere plays a central role in the production of energy from sunlight, as well as in the conversion of this energy by cellular respiration in the mitochondria, making it available for other biological processes. It is important for the function of cells and organisms that the reducing and oxidizing molecules are roughly in balance. This is known as redox balance. It is referred to as oxidative stress if this balance is disturbed, usually by an increase in oxidative processes [2,3]. The oxidative state is controlled and maintained by the cell’s own sensors, signaling pathways, and defense mechanisms, in which the transcriptional regulation of many antioxidative and cytoprotective enzymes by the NRF2–KEAP1 system, consisting of the redox state-sensing Kelch-like ECH-associated protein 1 (KEAP1) and the transcription factor nuclear factor erythroid 2 related factor 2 (NRF2), plays a central role [17,18].

### 2.1. Origin of ROS and Oxidative Stress

Oxidative stress occurs primarily when the amount of reactive oxygen species (ROS) exceeds the neutralization capacity. In addition to the superoxide (•O_2_^−^) and hydroxyl (•OH) radicals, these include hydrogen peroxide (H_2_O_2_) and singlet oxygen (^1^O_2_), as well as organic compounds [2,3]. A major source of ROS is the mitochondria, which are present in every cell and play a central role in the energy supply. ROS are formed during metabolic processes of the mitochondrial electron transport chain (“respiratory chain”), in particular the superoxide anion radical •O_2_^−^, H_2_O_2_, and the hydroxyl radical •OH. It is estimated that, in the mitochondrial respiratory chain, about 2% of the oxygen consumed is not converted to water but to superoxide radicals. Persistent oxidative stress may lead to the destruction of mitochondria, microfilaments, and proteins, which lose their function through oxidation, resulting eventually in an impairment of their function in metabolic processes.

Other important sources of ROS include the nicotinamide adenine dinucleotide phosphate (NADPH) oxidases (NOX) and metabolic processes involving, for example, heme-containing cytochromes such as detoxifying enzyme cytochrome P450 [1,3,4,19]. NOX enzyme complexes consist of several subunits and occur in several forms in different cell types [20]. They produce the superoxide radical from molecular oxygen, which, depending on the cell type or organ, is used not only to defend against pathogens but also as a signaling molecule. Accordingly, the NADPH oxidases are either located at cell membranes or at the membranes of specific organelles (phagosomes) of macrophages, neutrophil granulocytes, and dendritic cells of the immune system, where trapped microorganisms are killed [21].

In immune cells, as well as in many other cell types, reactive nitrogen-containing molecules, the gaseous free radical nitric oxide (•NO), play a role in addition to ROS. This is produced by three types of ubiquitously expressed nitric oxide synthases (NOS), which exist as endothelial (eNOS), neuronal (nNOS), and inducible (iNOS) isoforms [1,2]. While eNOS and nNOS are calcium/calmodulin-regulated enzymes, iNOS represents a cytokine-inducible form, which leads to a strong nitric oxide (NO) synthesis in immune cells (macrophage and microglia cells), as well as in other cell types, and it is involved in immune processes and controlled cell death. NO itself is an important messenger substance that, for instance, is involved in the regulation of blood circulation by vasodilation, neuronal functions, and immune defense. While it is not cytotoxic per se at normal concentrations, NO can react spontaneously with superoxide to form highly reactive peroxynitrite, which can damage the DNA and proteins, while it is also used in macrophages, for example, to defend against infections. In addition to the Fenton chemistry pathways, the peroxynitrite pathway poses a major oxidative stress-related threat to biological material.

Superoxide radicals can be converted to hydrogen peroxide (H_2_O_2_) by superoxide dismutases (SODs). This family of enzymes is, thus, the first antioxidative line of defense to control the superoxide radical (•O_2_^−^), a byproduct of oxygen metabolism or specifically produced in immune cells by NADPH oxidases [22]. With the participation of metal ions, they convert superoxide radicals to the less reactive H_2_O_2_. Superoxide dismutases occur in different variants in most living organisms and cell types and act in the cytoplasm, in the mitochondria, and in the extracellular space.

### 2.2. Protective Mechanisms

Although these reactive molecules can potentially cause damage to biological material and impede functionality, their presence and production should not generally be considered harmful. As indicated in some examples in the previous chapter, they are even indispensable for some biological functions and mechanisms [1,2,19,23]. For example, •NO and H_2_O_2_ are not only involved in the immune response, but also play a central role in the regulation of the redox state. H_2_O_2_ is also required for wound-healing processes or the correct formation of protein structures. It is important for the organism to keep ROS concentrations at a tolerable level, which is achieved through the cooperative action of antioxidants and enzymatic protection mechanisms, controlled by the NRF2–KEAP1 pathway, the key regulator of oxidative state and xenobiotic detoxification [17,18]. For example, provitamin A, vitamins C and E, and glutathione (GSH) act as antioxidants.

In addition, a number of enzymes play essential roles in controlling ROS. Peroxidases are able to process different forms of reactive peroxides, with H_2_O_2_ and lipid peroxides being the most relevant biologically in mammals. Different strategies and cofactors are used to neutralize these radicals by the addition of electrons. The peroxidase named catalase (CAT) plays a key role in the antioxidative defense system of many living organisms. It breaks down H_2_O_2_ to water and oxygen and, thus, neutralizes it [1,3]. CAT occurs in virtually all cell types and fulfils its function in specialized cell organelles, the peroxisomes, or in the cytoplasm and mitochondria. Peroxiredoxins (PRDx) also degrade H_2_O_2_, as well as organic peroxides [24]. Among other functions, for example, they regulate the cytokine-mediated signaling cascades and occur as antioxidant enzymes in mitochondria and in red blood cells. Glutathione peroxidases (GPx) and the GSH system are also vital. In humans and mammals, several types of glutathione peroxidases with preferences for either lipid peroxides or H_2_O_2_ have been identified [25]. The variants of GPx occur in specific cell types, as well as extracellularly or in the plasma. These enzymes can remove peroxides in a multistep process, converting reduced GSH into oxidized glutathione disulfide (GSSG). By the action of the glutathione reductase (GR), GSSG is then converted back to GSH, which is the predominant form of glutathione and an important antioxidant under physiological conditions.

### 2.3. Detection of Oxidative Stress

Intracellular ROS concentrations depend on the balance between ROS generation and its elimination. In general, fluctuations in ROS production and the rapid response of the related protective mechanisms can be measured. Several experimental approaches have been described to detect ROS generation, with dyes turning fluorescent upon contact with ROS being the one that is most commonly used [26]. However, it has to be noted that specificity and sensitivity for a particular ROS species are limited, depending on the method and compound applied. The activity or amount of superoxide dismutases (SODs), catalases (CATs), or peroxidases can also be used as an indicator of oxidative stress. An important and frequently used biomarker for oxidative stress is the availability of GSH or, rather, the ratio of reduced to oxidized glutathione (GSH/GSSG). The activity of glutathione reductase also provides information on the redox state.

In addition to direct measurements of ROS production and the antioxidative defense process, damage to biomolecules or their degradation products can be detected, especially as indicators for sustained oxidative stress. An increase in oxidized bases in the DNA (i.e., 8-oxo-G/8-OHdG) and the carbonylation of proteins serve as surrogate markers for ROS. Malondialdehyde (MDA), a degradation product of unsaturated fatty acids, is also a frequently analyzed biomarker for oxidative stress [27]. Malondialdehyde is formed during normal enzymatic reactions, as well as by ROS-induced peroxidation of membrane lipids (lipid peroxidation). MDA itself is highly reactive and can lead to structural changes and damage to DNA and proteins. Elevated MDA levels are observed in many chronic diseases, and such pathological levels may contribute to a variety of long-term health impairments.

## 3. Impact of EMF on the Nervous System

Due to their longevity and limited renewal, neurons are considered particularly sensitive to oxidative stress. Oxidative stress caused by chronic inflammation may result in substantial cell damage. Thus, ROS formation and consistent oxidative stress have been associated with neurodegenerative diseases and aging [1,20,23], whereby—among many other factors and environmental influences—an involvement of EMF-induced oxidative stress is conceivable. On the other hand, many aspects of neuronal development, plasticity, and signal processing rely fundamentally on the formation of ROS to establish and ensure normal functionality [19,23,28]. Thus, temporal changes of ROS formation in brain cells do not necessarily have to result in negative and health-relevant effects.

### 3.1. Observations in EMF-Exposed Animals

After short- or long-time EMF exposure, ROS production and the related antioxidant defense systems have mostly been investigated in laboratory animals, namely rats and mice (Supplementary Materials, Tables S1 and S3). In addition to the basic question of whether EMF exposure causes oxidative stress, in some cases, information about its transient or permanent nature, requiring ROS measurements in several animal groups with different exposure durations, provided additional data with respect to health impact. However, well-founded conclusions on health impact are only possible if additional functional investigations, such as learning behavior or the occurrence of DNA damage, are also measured. Small group sizes, from five animals upward, are considered meaningful studies with experimental animals.

In the last decade, about 50 original studies in laboratory animals have been published on EMF exposure and oxidative stress in the brain. In a comprehensive work with Sprague-Dawley rats, increased ROS activity or formation of MDA, 8-OHdG, and serum nitrite was observed after 6 months of RF-EMF exposure at different frequencies (900, 1800, and 2100 MHz) for 2 h per day [29]. The whole-body specific absorption rate (SAR) of 0.174–0.638 W/kg was below the existing regulatory limits and recommendations. Concurrently, indications for increased DNA damage were found in the brain, which correlated with the applied frequency but was only significantly different from the sham controls at 2100 MHz. At the same time, the capacity of the antioxidative protection system was exhausted as the measured antioxidative markers were significantly lower compared to sham-exposed animals [29]. These results indicate that oxidative stress induced by RF-EMF can lead to DNA damage in neurons during prolonged exposure of the animals. Virtually identical results were also found in several other studies [30,31,32,33,34]. In the study by Megha et al., Fischer-344 rats were exposed to RF-EMF with frequencies of 900, 1800, and 2450 MHz at whole-body SAR values of 0.59, 0.58, and 0.66 mW/kg, respectively, for 60 days (2 h/day and 5 days/week) [32]. Biomarkers for oxidative stress (including MDA) and various inflammatory markers were augmented correlating with the increasing frequency, while the antioxidative activity (SOD, GSH) decreased [32]. Similar observations were reported by Sahin et al., who measured increased ROS production in the brain of Wistar rats after universal mobile telecommunications system/third generation (UMTS/3G)-modulated RF-EMF exposure (2100 MHz, whole-body SAR: 0.4 W/kg; 6 h/day and 5 days/week) [33]. However, this ROS increase occurred only after 10 but not after 40 days of exposure, correlating with DNA damage but decreased lipid peroxidation in brain cells [33]. The absence of DNA damage after 40 days may indicate adaptation to exposure or enhanced capacity of DNA repair. Evidence for adaptation to or recovery from induced oxidative stress by 900 MHz RF-EMF (2.5 mW/cm^2^, 1 h/day) was also observed in male Sprague-Dawley rats. ROS levels were elevated in the brain after 60 days of irradiation. However, ROS levels were not different from controls after a regeneration phase of 30 days without irradiation [35]. Correlating with exposure duration, changed levels of DNA damage were also found in hippocampal cells after 900 MHz RF-EMF exposure [34]. RF-EMF exposure for 90 days (1–4 h/day, 5 days/week at 0.231 W/kg) increased ROS formation, reduced antioxidative markers (SOD and CAT), and induced the formation of inflammatory cytokines. In addition, neuronal cell degeneration and other morphological changes in the brain were observed [34]. In contrast, an increase in oxidative stress was induced by RF-EMF exposure without affecting DNA damage levels in some other studies [36,37,38,39].

Adding to the studies with functional aspects, descriptive studies including analyses of ROS with and without measurements of antioxidative biomarkers were also published. With respect to health effects, they are less conclusive, especially if no data are available on whether the observed effects are temporary or persistent. Nevertheless, most of the studies indicate changes in ROS formation and/or oxidative stress biomarkers [35,40,41,42,43,44,45,46], examining and demonstrating morphological changes of the brain tissue in some cases [35,42,43,46,47,48]. The study by Kesari et al. described an increase in ROS formation and an elevation of oxidative stress markers, a marked reduction of antioxidative markers, and increased apoptosis rates in the brain of Wistar rats exposed daily for 2 h to 900 MHz RF-EMF for 45 days (pulsed at 217 Hz; SAR: 0.9 W/kg) [49]. The effects of exposure on this readout were measured once after an exposure period of 45 days, suggesting that prolonged RF-EMF exposure did not lead to exhaustion of ROS production and/or adaptation in this case. Similarly, RF-EMF (915 MHz, 0.79 mW/cm^2^) exposure of male Wistar rats for 1 h/day for 1 month resulted in increased oxidative stress and NO formation and reduced antioxidant markers [50]. These purely descriptive studies have limitations, especially when a cell phone was used for exposure, dosimetry was missing, and/or no SAR value or dose was provided. The marker for ROS-related DNA damage, 8-oxo-G, was also increased after RF-EMF exposure (2.45 GHz, whole-body SAR: 0.2 W/kg for 30 days and 1 h/day) in rat brains, while oxidative protein products were not altered [44]. Again, this is a descriptive study, which focused on possible antioxidant effects of garlic extracts, similar to a second study by another group [41], in which RF-EMF (1.8 GHz, whole-body SAR: 0.4 W/kg, 1 h) after 3 weeks of exposure showed an increase in protein oxidation, as well as more NO in the brain. Lipid peroxidation in the brain was found at whole-body SAR values in the range of 0.1–0.3 W/kg [48].

Shahin et al. (2017) also found an increase in ROS and associated changes in the antioxidative defense system in the hypothalamus of female Swiss mice exposed to 1800 MHz RF-EMF for 100 days with no SAR value reported [51]. The same group reported changes in stress-related hormones and associated markers in the hippocampus of male Swiss mice exposed to 2.45 GHz RF-EMF at 0.0146 W/kg SAR for 15, 30, or 60 days [52]. This stress, probably associated with induced NO production and signaling, led to reductions in learning and spatial memory performance of these mice. Long-term exposure for 8 months at 1950 MHz (SAR: 5 W/kg, 2 h/day for 5 days/week) revealed no remarkable differences in programmed cell death, oxidative stress, apoptosis, genotoxicity, and motor activity in 14 month old female mice (C57BL/6J) compared to controls [37]. An increase in oxidative stress was observed in the animals due to age, but RF-EMF exposure did not induce oxidative stress, and the movement behavior of the animals was not affected.

An increase of ROS markers compared to sham-exposed and cage controls was also seen in the spinal cord of very young and middle-aged Sprague-Dawley rats after 900 MHz RF-EMF exposure (1 h/day, calculated whole-body SAR: 0.01 W/kg) for 25 days [53]. Interestingly, biomarkers for antioxidative activity were elevated, indicating that the capacity of the antioxidant system was not yet exhausted and presumably able to counteract ROS formation. Nevertheless, morphological alterations of the spinal cord, such as tissue loss, vacuolation, and changes in myelin integrity, were observed, which might compromise proper neural signal transmission. Such changes, particularly demyelination and scarring of the myelin sheath, occur, for example, in multiple sclerosis. Changes in neurochemical parameters, as well as pathophysiological damage caused by inflammatory processes in various brain regions (i.e., hippocampus and cortex), are usually associated with reduced memory performance, DNA damage, and/or apoptosis. Correlating with the frequency of the radiation, such effects were reported by Megha et al. upon low-intensity RF-EMF exposure (whole-body SAR: about 0.6 mW/kg) [32]. They provided scarce information on the dosimetry, and the actual exposure in the brain is likely to be different from the estimated whole-body SAR values. However, an increased ROS production was found in the brain of rodents at higher SAR values (>1 W/kg) [35,43,54,55]. Ertilav et al. reported an increase in ROS in hippocampal neurons as well as in dorsal root ganglia after RF-EMF exposure of young female Wistar rats [43]. Rats were exposed to 900 or 1800 MHz RF-EMF with 217 Hz pulses for 12 weeks (1 h/day, 5 days/week) at an average whole-body SAR of 0.1 W/kg (local SAR ranging from 0.01–1.1 W/kg with the highest values for the head region). Transient receptor potential cation channel subfamily V member 1 (TRPV1) currents, intracellular calcium concentrations, mitochondrial membrane depolarization, and apoptosis were also significantly enhanced in neuronal cells of exposed animal in a frequency-dependent manner [43]. These observations are potentially relevant due to the role of spinal ganglia the hippocampus in pain transmission and behavior, as well as cognitive functions, although no functional experiments on pain or memory performance have been performed. Neither measurements nor calculations of SAR levels in the brain and spinal cord have been presented and, therefore, the level of exposure of these tissues remains unclear.

An impairment of learning behavior and memory performance by exposure was observed in other studies [56,57,58]. Tang et al. reported a reduction in memory performance in male Sprague-Dawley rats after 900 MHz RF-EMF exposure (whole-body SAR: 0.016 W/kg, brain SAR: 2 W/kg) for 28 days, associated with changes in the activity of the mitogen-activated protein kinase signaling pathway (mpk-1, external signal-regulated kinase (pERK)) [57]. Similarly, cognitive performance of Fischer-344 rats was decreased after a 900 MHz RF-EMF exposure for 30 days (whole-body SAR: 0.0058 W/kg), which was associated with increased oxidative stress and inflammatory markers in the brain [56]. Exposure to 1500 MHz RF-EMF decreased SOD levels in the brain of Wistar rats, correlating with neural toxicity and changes in learning and memory performance [58]. Thus, the findings of these studies suggest that increased formation of ROS by RF-EMF exposure is associated with an impairment of cognitive abilities.

There have been only a few studies in Wistar rats exposed to a Wireless Fidelity (WiFi) signal (2.45 GHz) [50,59,60,61]. Othman et al. found impaired neurodevelopment in offspring during the first 17 postnatal days, an increase in cerebral ROS and lipid peroxidation on postnatal day 28 but not 43 after prenatal exposure for 2 h/day, and decreased antioxidant levels (CAT, SOD) [61], indicating an exhaustion of the antioxidative capacity in the brain. In a study from the same group, prenatal WiFi exposure in combination with physical constraint was associated with increased anxiety behavior, motor deficits, and impaired exploratory behavior in adult male rats. Restrained animals, WiFi-exposed rats, and a combination of both resulted in increased oxidative stress in the brain in both sexes [60]. WiFi exposure of adult male Wistar rats alone or with physical constraint impaired the learning behavior and memory performance accompanied by an oxidative stress response in the brain. [59]. Albeit having some methodological shortcomings, the study by Asl et al. also showed increased ROS and NO production in rats exposed to RF-EMF/WiFi (2450 MHz; 0.98 mW/cm^2^) [50].

In the context of neurological disorders, the immediate response to short-term RF-EMF exposure was also assessed. In a mouse model with chemically induced epilepsy, the influence of RF-EMF on oxidative stress caused by epilepsy was investigated, exposing them (900 MHz, SAR: 0.3 W/kg) for 15 and 30 min before and/or after induction of the epileptic seizures [62]. While the antioxidant activity was significantly reduced, markers for ROS and lipid peroxidation in the brain were induced, whereby the time-point of RF-EMF exposure was not pivotal for the observed effects. In an Alzheimer’s disease model, the stress marker cortisone and markers for oxidative stress in the brain of rats after RF-EMF exposure for 15 min (1.5, 6 W/kg) and for 45 min (6 W/kg) were measured, coincidental with the assessment of memory performance. While oxidative stress in the brain increased, cortisone levels and memory performance of RF-EMF-exposed Alzheimer’s animals decreased markedly, an effect that was not observed in wildtype (devoid of Alzheimer) animals [63]. This study indicates that animals with a prior neurodegenerative condition might be more sensitive to RF-EMF exposure.

Compared to neuronal effects of RF-EMF, fewer studies have been published for the low-frequency range in the last decade. A dose-dependent increase in ROS, lipid peroxidation, and decreased antioxidant defense were observed in different brain regions of young male Wistar rats continuously exposed to 50 Hz ELF-MF (50 and 100 µT) for 90 days [64]. More pronounced at the higher field strength, the production of ROS was also increased and the antioxidant response decreased after ELF-MF exposure (100 and 500 µT, 50 Hz) of male Sprague-Dawley rats for 2 h/day for a total duration of 10 months [65]. Similar results were obtained in a study with shorter exposure duration. In male rats exposed to ELF-MF (500 µT, 50 Hz) for 7 days, ROS was increased in various areas of the brain and increased lipid peroxidation and activity of the protective antioxidative mechanisms were observed [66].

At higher magnetic field strengths (2.3 mT), increased ROS production was observed in the cerebellum of male mice (Balb/C) after a short exposure (3 h) with 60 Hz ELF-MF, while some antioxidative markers were elevated (SOD, ascorbic acid) and others remained unchanged (GSH, GPx) [67]. Apparently, antioxidative processes are initiated after such a short exposure period. However, it is not expected that the antioxidative system is already exhausted or impaired, indicating a state of oxidative stress as in studies with longer exposure. For example, ROS production, as well as lipid peroxidation, in the brain of young male Sprague-Dawley rats was altered after 40 Hz ELF-MF exposure (7 mT), depending on the duration of daily exposure (30 versus 60 min) for 10 days [68]. In this situation, 30 min of daily exposure was sufficient to increase lipid peroxidation, while detectable ROS formation required 60 min exposure, suggesting a threshold for exposure duration or cumulative dose.

As mentioned before, confinement stress caused by animal exposure in tubes can lead to systemic oxidative stress. In the study by Martinez-Samarano et al., an alteration of various biomarkers for oxidative stress (SOD, CAT, NO) and increased ROS levels were measured in the brain of male rats after acute 60 Hz ELF-MF exposure for 2 h (2.4 mT), whether in cages or in tubes [69]. SOD levels were significantly lower in restrained ELF-MF-exposed animals compared to animals in cage controls and the respective sham controls. CAT levels were reduced in ELF-MF-exposed animals in cages when compared to the sham control, but a difference in CAT levels was found when the rats exposed in tubes were compared to the corresponding sham control. NO levels were significantly lower in rats exposed to ELF-MF in tubes compared to all other groups. These data show that ELF-MF induces an adaptive response even at short-term exposure, leading to activation of protective antioxidative measures. The stress hormone cortisone was elevated only in control animals that spent time in tubes, while ELF-MF exposure did not alter the outcome [69]. There are also indications of inflammatory response in the brain. NO was elevated in various brain regions of male Sprague-Dawley rats exposed to a 60 Hz ELF-MF (2 mT) for 5 days, which was supported by an increased level of nNOS [70]. Nevertheless, the number of neurons remained unchanged and ultrastructural examinations of the mitochondria did not reveal any differences compared to the controls. As NO can react with superoxide, this could then lead to damage of DNA and proteins, depending on its extent. However, no further investigations in this direction were performed, precluding a firm statement about damage to both biomolecules by ELF-MF.

In addition to duration and dose of exposure, the age of the animals is also a factor influencing the defense mechanisms against stress factors since defense and counter-regulatory mechanisms decrease with age [71]. In line with that notion, Falone et al. showed that the extent of antioxidative defense mechanisms in the cerebral cortex of female Sprague-Dawley rats depended on age, regardless of exposure [72]. The antioxidative capacity was less efficient in 19 month old animals compared to 3 month old animals, yet an influence of 50 Hz ELF-MF exposure (100 µT) for 10 days on the antioxidative activity was observed overall. CAT activity was significantly decreased and SOD and GSH reductase were increased in the young rats after ELF-MF exposure. In young animals, this was accompanied by signs of increased neuromodulation (elevated levels of nerve growth factor NGF and tropomyosin receptor kinase A TrKA). Such neurotrophins cause targeted connections between neurons and lead to activation of cellular signaling pathways, which may ultimately result in an antiapoptotic effect. In contrast, older rats were not able to stimulate such protective processes, and a marked reduction in antioxidative parameters was found in the brain [72,73]. These results suggest that EMF might be a risk factor in older individuals due to their reduced capacity of antioxidative defense.

Environmental cofactors may also modulate the occurrence and response of oxidative stress. In the brain of Kumming mice, the effect of aluminum with and without 50 Hz ELF-MF irradiation (2 mT) for 6 days/week and 8 weeks on the occurrence of oxidative stress, as well as Tau and phosphorylated Tau proteins, was investigated. The Tau protein is important in neurodegenerative syndromes such as Alzheimer’s disease, as it binds to microtubules in cells, regulating their cohesion. ELF-MF exposure caused an increase in ROS and a reduction in the measured antioxidative biomarkers, while the additional administration of aluminum did not promote any further impairment [74]. Structural abnormalities, a reduction in the number of neurons, and changes in the phosphorylated form of Tau at S404 and S396 indicated neurodegenerative effects of subchronic ELF-MF exposure, which was supported by the impairment of learning and memory performance in ELF-MF-exposed animals.

Overall, the studies related to RF-EMF and ELF-MF show that various factors are influencing the response to EMF exposure. In addition to duration and dose of exposure, adaptive processes and age-related capacities to respond to oxidative stress are of central importance.

### 3.2. Observations in EMF-Exposed Cultured Neuronal Cells

In support of the findings in animals, EMF-induced oxidative stress was also most frequently investigated in cultured cells of neuronal origin (Supplementary Materials, Tables S2 and S4). In the last 10 years, more than 30 manuscripts have been published, in which, among other endpoints, the influence of EMF on the formation of radicals and ROS or biomarkers for oxidative stress was analyzed, about half in the low- and half in the radio-frequency range. The cell models used were largely tumor cells of neuronal origin (neuroblastoma: SH-SY5Y, NB69, Neuro-2a; glioma: U-87MG, C6; pheochromocytoma: PC12), in addition to established cell lines (HT22) and primary neurons of the brain, as well as astrocytes from humans and rodents.

The influence of ELF-MF was mainly investigated in tumor cell lines, where exposure was frequently found to influence ROS formation or markers of oxidative stress and to lead to changes in the antioxidative defense system. It is important to note that tumor cells often have an intrinsically disturbed oxidative balance and may, therefore, react differently to EMF or other treatments than a normal cell. However, primary neurons from the brain also reacted to repeated 50 Hz ELF-MF exposure at a flux density of 2 mT by an increased production of ROS, an upregulation of the NADPH oxidase NOX2, and faster neuronal cell death, especially pronounced in older cell cultures [75]. This indicates that the findings from the experiments with tumor cells are at least partially transferable to normal and immortalized cells. For example, slightly elevated values for superoxide and H_2_O_2_ were found in SH-SY5Y neuroblastoma cells [76,77,78] when exposed to a 1 mT field for 1–3 days. In addition, the increase in ROS was attenuated by SOD administration [79] and changes in various markers of oxidative stress (CAT activity, oxidative protein modification) were observed. On the other hand, the increase in ROS appeared to be more pronounced when acute cell responses, about 1 to 6 h after exposure start, were evaluated [78,80]. Simultaneously, an increased activity of the NO synthase was observed, which might indicate a function of ROS and NO as a signaling molecule in this context. In fact, there is evidence for ROS-mediated alteration of cellular signaling pathways by ELF-MF exposure from studies with neuroblastoma cells [77,81]. In addition, it was found in PC-12 tumor cells that a short ELF-MF exposure of 30 min (0.1 and 1 mT) induced a differentiation process mediated by a rapid increase in ROS formation [82]. This increase in ROS did not occur when the cells were advanced in the differentiation process or exposed for a longer period of time [82,83]. Similar mechanisms involving ROS formation as a signaling molecule seemed to work when mesenchymal stem cells from human bone marrow were differentiated into neural cells under 50 Hz ELF-MF exposure (1 mT) [84,85]. Here, the efficiency and proportion of differentiation to the distinct neural cell types was altered by exposure, most likely because the increased ROS formation triggered or modified signaling cascades.

It is possible that a constant stimulation of ROS formation cumulatively boosts the antioxidative defense systems. Therefore, after short exposures, little or no evidence of antioxidative stress markers, such as the ratio of GSH/GSSG, can be detected [86], while, after longer exposure, increases in these markers, as well as changes in cell response to additional stress, were observed [76,87]. In this context, it is also worth mentioning that similar observations were also made for weaker ELF-MF (≤100 µT) in combination with other triggers of oxidative stress, whereby the cellular adaptations and consequences were still detectable for a prolonged time [88,89,90,91]. Hence, there is quite consistent evidence that exposure to 50 Hz ELF-MF leads to increased formation of ROS in cultured cells of neuronal origin. As a consequence, activation of a variety of cellular regulatory mechanisms triggers corresponding cell responses, whereas it may also lead to persistent oxidative stress situations.

Similar observations were made in RF-EMF-exposed neuronal cells, although the findings were less consistent and partly even contradictory. This could also be due to the technically and dosimetrically more demanding implementation in this frequency range and the diversity of the investigated RF-EMFs with respect to dose, carrier frequencies, inclusion of signal modulation, etc. For example, in isolated rat neurons exposed to a 1.8 GHz global system for mobile communications/second generation (GSM) signal for 24 h, increased ROS formation, in addition to signs of DNA damage and reduced functionality of mitochondria, was found at 2 W/kg SAR [92], whereas this increase was only significantly detectable at 4 W/kg SAR in another study [93]. In isolated astrocytes of humans, mice and rats, however, there was no evidence of an increase in ROS by 900 MHz GSM signals for 24 h, and there was even less ROS produced in the mitochondria (SAR: 0.2 W/kg) [94]. Similarly, no signs of inflammation, such as more iNOS or NO formation, were found in astrocytes after exposure to 1.8 GHz RF-EMF for 1–24 h (SAR: 1 W/kg) [95], although an acute increase in ROS after 20 min of exposure to modulated but not unmodulated 900 MHz RF-EMF was found [96]. On the other hand, in a mouse neuronal cell line, exposure to a 1.95 GHz RF-EMF (UMTS/3G signal) showed marginal effects on ROS formation and other parameters on its own, but differentially modulated signaling pathways and cytotoxicity when ROS formation was triggered by glutamate or β-amyloid [97,98].

A series of studies have been conducted with neuronal tumor cells. No increase in ROS formation was found in SH-SY5Y neuroblastoma or U-87MG glioma cells after acute exposure to 872 MHz RF-EMF (GSM signal or carrier wave, SAR: 5 W/kg) [99,100], a 900 MHz GSM signal (SAR: 4 W/kg, 2 min on/off) [101], a combination of modulated 867/1950 MHz RF-EMFs (SAR: 4 W/kg) [102], and a 1.8 GHz GSM signal (SAR: 2/10 W/kg) [103]. In short-term co-exposure experiments, the effect of an ROS-triggering substance such as menadione and H_2_O_2_ was amplified by these RF-EMFs [99,102]. In contrast, these cell types reacted to a non-modulated 1.8 GHz RF-EMF and comparable exposure duration with the formation of ROS, oxidative protein modification, lipid peroxidation, and alteration of the antioxidative defense system (GSH) [104,105]. Similar to the observations for ELF-MF, the ROS increase seems to be more prominent after short rather than after continuous (≥12 h) RF-EMF exposure [94,103,105]. For prolonged exposure, there are once more indications for a boost of the antioxidative defense system, an impact on mitochondrial function and autophagy activity [101,106], and even an accumulation of DNA damage and cell death [92,105,107].

### 3.3. Assessment of EMF-Induced Oxidative Stress in the Nervous System

In general, a distinction must be made between studies that are purely descriptive and those that simultaneously investigate functional effects, such as learning and memory performance. The latter ones provide more information on a possible health-relevant impact on the animals due to increased oxidative stress caused by EMF exposure. It is also important to note that, for the assessment of health relevance, ROS formation and temporary oxidative stress are not harmful per se [1,3,4,23]. These reactive molecules are also part of physiological processes and fulfil functions, for example, in the immune response or the correct formation of protein structures. Damage with possible health relevance only occurs if the redox equilibrium, which is controlled and maintained by sensors, cellular signaling pathways, and protective antioxidative mechanisms, is disturbed over a long period of time, either permanently or repeatedly. If the latter is the case, various physiological processes such as cell proliferation, neuronal differentiation and activity, and development are affected. ROS formation and decreased antioxidative counter-regulation also occur in aging processes. Therefore, models investigating the influence of EMF exposure on the redox system are of interest and importance for a possible impairment of old individuals or those with pre-existing damage (neurodegeneration). Oxidative stress is the cause and/or consequence of neurodegenerative syndromes such as Alzheimer’s and Parkinson’s disease, which are accompanied by reduced learning and memory performance.

An increased occurrence of ROS, as well as the burdening and exhaustion of antioxidative mechanisms after exposure to different EMF in the radiofrequency range and SAR, even at values below the recommended regulatory limits, and damage to the DNA were associated with prolonged exposure over weeks or months, applied in many cases only for a few hours per day [29,30,31,32,33,34]. However, one study also reported that recovery and a return to normal values after the end of exposure occurred [35].

Studies on mechanisms such as those related to calcium channels are particularly informative as calcium concentration-dependent cellular responses may result in a multitude of pleiotropic effects [9]. Voltage-gated calcium channels were shown to be activated by nonthermal pulse-modulated 27 MHz RF-EMF, leading to an increase in NO [108] while nonselective calcium channels such as transient receptor potential (TRP) channels are activated by oxidative stress [109,110]. For instance, the TRPV1 channel, belonging to the calcium-permeable TRP superfamily, can be activated not only by stimuli such as heat and capsaicin, but also by oxidative stress. Activation of TRPV channels by oxidative stress/EMF was demonstrated to result in an increase in neuronal calcium concentrations that may lead to physiological changes and pathological processes such as apoptosis [43,111,112]. The occurrence of TRPV1 is particularly high in neurons of the hippocampus and in spinal ganglia, where it is probably involved in the transmission of pain, which is impaired in neurodegenerative processes [109,110].

In part, the changes in redox balance were accompanied by morphological changes that resemble those in neurodegenerative diseases [34,35,42,43,46,47,48,53]. In general, studies, in which ROS, antioxidative markers, learning behavior, and memory performance were examined, suggest an impairment of neuronal functions of the animals. Thus, there is evidence that, at least in animal models, increased ROS production by EMF is associated with an impairment of cognitive abilities [51,52,56,57,58,59,60]. Notably, RF-EMF exposure affected the memory performance of animals with neurodegenerative pre-damage of the brain (i.e., Alzheimer’s disease model) more than in control wildtype animals [63], indicating an enhancement of conditions of impaired learning behavior by RF-EMF. In addition to such pre-existing conditions, other environmental or risk factors may also play a role in whether and to what extent oxidative stress due to EMF exposure occurs. There is evidence that age is such a risk factor [72]. Due to their reduced antioxidative capacity in the brain, older individuals are less efficient in compensating for increased ROS formation, and adaptive processes are exhausted more quickly than in young individuals [71]. Newborns are also more vulnerable to oxidative stress, as antioxidative protection mechanisms are not fully developed in the first days or weeks of life, depending on the species.

Methodological factors must also be taken into consideration when evaluating experimental studies. Often, RF-EMF exposure was performed in carousel exposure systems, in which the animals are placed into narrow tubes during exposure, facilitating a homogeneous and defined exposure. However, this procedure provides a source of error if the animals are not trained in advance, since restraint stress can also lead to oxidative stress [69]. In these experiments, sham controls and prior training of the animals to the conditions of exposure are important. In addition to increased ROS production, a change in the anxiety behavior, but not in memory performance, was found after WiFi exposure of rats exposed in tubes, which was enhanced by the exposure [59,60].

ROS formation and impairment of antioxidative protection measures by EMF were demonstrated in cell studies with neurons or neural cells, which aim at understanding the mechanisms underlying the observations in animal models. There is consistent evidence that cellular signaling pathways regulated by ROS are affected [77,81,82,97,98]. The extent of induction, as well as the possibility of counter-regulation has to be considered, presumably requiring a threshold level or persistence to translate to health impairment. It seems that the degree of cell differentiation or age is critical; cells that were further differentiated generally were less sensitive compared to undifferentiated cells or cells in an early stage of differentiation [75,82,84,85]. It is noteworthy that the induction of ROS and signs of oxidative stress appear to be more reproducible in neural cells exposed to ELF-MF than to RF-EMF [75,76,77,78,79,80,81,82,92,93,94,96,97,98,99,100,101,102,103,104,105,113]. Higher doses of RF-EMF exposure mostly resulted in more pronounced effects, although not consistently, and a temperature increase or other confounders cannot always be excluded [92,93,96,99,100,101,102,103]. Other methodological factors, such as keeping sham controls in a separate incubator, pose a risk of false-positive findings [114,115]. For example, vibrations, as well as EMF of the incubator or its inadequate shielding, come into play, and it cannot be excluded that these factors influenced the measurement parameters recorded in some studies. The duration of exposure seems to play a role, whereas a shorter exposure for few hours rather than prolonged ones led to increased ROS production and a temporal reduction in antioxidative processes [78,80,94,96,103,105].

## 4. EMF Effects on the Blood and Immune System

The influence of technology-related EMF on cells of the immune system has also been a frequent topic of investigation in recent years [8,11,12]. The functioning of the immune system is inseparably connected to the formation of ROS and NO. ROS and NO play a vital role in the elimination of foreign or damaged cells by phagocytosis and are involved in the inflammatory reaction and activation of the immune response [2,21]. In this respect, it is conceivable that suppression, as well as a constant activation, of these processes by EMF could lead to impairment of health. Therefore, the influence of EMF on various aspects of immune responses and the development of hematopoietic cells and the microglia cells as a functional equivalent in the central nervous system have been studied (Supplementary Materials, Tables S1–S4).

While several publications on oxidative stress and EMF exposure in isolated and cultured blood and immune cells are available, the number of animal studies is limited, whereof only some of them provided information on ROS markers in blood.

### 4.1. Oxidative Stress in EMF-Exposed Animals

Exogenous influences, such as stress, can alter the organism’s response to subsequent stimuli. In a short-term study, mice were exposed to 900 MHz RF-EMF (SAR: 0.5 W/kg) 4 h/day for 1 week before treating them with the cancer medication bleomycin [38]. This substance acts by oxidation of molecules, leading to oxidative stress, and causes DNA damage among other implications. Interestingly, leukocytes of RF-EMF-exposed animals were less damaged by bleomycin compared to controls, and ROS was decreased in the plasma and some tissues, while SOD was increased in the lung [38]. These findings suggest that RF-EMF exposure may cause systemic changes, which in turn affects the cellular response to other stressors. This phenomenon is known as “adaptive response”, which is likely to play an important role in real life situations where many stress and environmental stimuli occur simultaneously. Related findings were obtained in young and adolescent Wistar rats after 900 MHz RF-EMF exposure (SAR: 0.28–0.78 W/kg) [116]. ROS and oxidative stress markers were measured directly after 45 days of exposure for 2 h per day or following a recovery period of 15 days [116]. This approach facilitates the determination of postexposure persistence and the ability of the organism to counteract the oxidative stress. RF-EMF increased the antioxidative activity in all lymphoid organs regardless of the age, and that the recovery period was insufficient to return to normal SOD levels when exposure was started at 2 weeks compared to the animals at 10 weeks of age. As oxidative stress biomarkers were elevated in all animals after RF-EMF exposure, this difference may originate from the fact that the enzymes of the protective antioxidative defense system are not yet fully developed or present in the very young rats. Depending on the marker, the normalization in the recovery phase was more successful in the 10 week old rats. In most of the lymphoid organs, as well as in plasma and lymphocytes, increased lipid peroxidation was seen directly after exposure and at the end of the recovery phase [116]. This comprehensive and well-documented study shows, on the one hand, that the oxidative stress situation may persist for a longer period of time and, on the other hand, that very young individuals are less able to compensate for the increase in ROS.

In Wistar rats, similar results were demonstrated, including increased lipid peroxidation due to exposure to 2.45 GHz WiFi-like signals for 35 days (50 Hz pulses, whole-body SAR: 0.14 W/kg) in the spleen [48] and for 28 days (217 Hz pulses, SAR: 0.143 W/kg, 45 min/day) in plasma and erythrocytes [117], accompanied by a reduced activity of antioxidative markers. Furthermore, oxidative DNA (8-oxo-G) and protein products, indicating oxidative stress, were elevated in plasma cells of rats after a daily 1 h exposure to 2.45 GHz RF-EMF (whole-body SAR: 0.2 W/kg) for 30 days [44]. Signs of oxidative stress were described in RF-EMF-exposed mice. Changes in ROS and enzymes of antioxidative defense (SOD, CAT, glutathione *S*-transferase (GST)) were found in blood, as well as in the liver, kidneys, and ovaries, of pregnant Parkes mice exposed to 2450 MHz RF-EMF (SAR: 0.023 W/kg) for 45 days [118]. The same group also reported changes in stress-related hormones and associated markers in the blood of Swiss mice exposed to 2450 MHz RF-EMF (SAR: 0.0146 W/kg) for 15, 30, or 60 days [52]. In contrast, no effects on oxidative stress, lipid peroxidation, or elevated NO levels were measured in the blood serum of Wistar rats upon exposure to 1.8 GHz RF-EMF (whole-body SAR: 0.4 W/kg) daily for 1 h for 3 weeks [41]. Likewise, lipid peroxidation and reduced GSH were not increased in the blood of rats that were exposed to UMTS-modulated RF-EMF daily for 40 min for 2 weeks [119]. However, the animals were exposed using a cell phone in talk mode in their cages, which is associated with large fluctuations and uncertainty regarding exposure dose.

Recent animal studies on oxidative stress induced by exposure to 50 Hz ELF-MF are scarce. Exposure for 10 months at a field strength of 100 µT to male Sprague-Dawley rats resulted in modifications of DNA bases (8-oxo-G and others) in white blood cells, which are produced by oxidative processes and may be mutagenic [120]. Interestingly, these effects were no longer observed at a higher field strength of 500 µT. However, no direct conclusions can be drawn, since DNA damage was not investigated. Increased ROS production and lipid peroxidation were measured in the plasma of female rats after ELF-MF (50 Hz, 100 µT) exposure for 3 h/day. These effects seemed to be cumulative with duration of exposure, being stronger for 100 days than for 50 days of exposure [121].

### 4.2. Radical Formation in EMF-Exposed Cells of the Blood and Immune System

Cancer cell lines were used as a model system in the majority of in vitro studies, e.g., various myeloid leukemic cells such as THP-1 monocytes, K562 myelocytes, NB-4 and HL-60 promyelocytes, and RAW 264.7 macrophages. In addition, established microglia cells (human: HMO6, CHME-5; mouse: N9) and isolated hematopoietic stem cells, monocytes, macrophages, and T cells from humans and mice were employed (Supplementary Materials, Tables S2 and S4).

An increase in superoxide formation was observed in K562 leukemic cells after 50 Hz ELF-MF exposure for 1 h with relatively low flux densities (25, 50, 100 µT) [122]. In this cell system, the time of analysis also seems to play a role. A transient stimulation of CAT activity and a time window for increased production of superoxide and iNOS were found for ELF-MF exposure (1 mT) [123]. In this case, the superoxide was generated by cytochrome P450 enzymes, which are phase I enzymes that play an important role in biotransformation of substances, including food components and pharmaceuticals. Exposure also altered the cell response to the tumor promoter phorbol-12-myristate-13-acetate (PMA) that triggers cell differentiation processes involving ROS. Prolonged or repeated ELF-MF exposure, however, provided little evidence for oxidative stress and ROS formation, although here an influence of exposure on cell responses to other factors was also found [124,125]. Accompanied by stimulation of ROS formation, on the other hand, prolonged 50 Hz ELF-MF exposure (2 mT) enhanced the differentiation of NB-4 promyelocytic leukemia cells by all-*trans*-retinoic acid (ATRA) but not PMA [126].

There have also been some studies, which looked at effects of ELF-MF on phagocytosis and immune function. For example, it was found in the THP-1 human monocyte leukemia cell line that ELF-MF exposure (1 mT) led to increased iNOS activity and NO production, whereas the activity of the antioxidative enzymes SOD and CAT were reduced [127,128]. In both cases, additional exposure led to changes in the immune response triggered by staphylococci or lipopolysaccharides (LPS). In this regard, 50 Hz ELF-MF exposure modulated the cellular response to the LPS treatment and underlying signaling cascades, involving the antioxidative heme oxigenase-1 (HO-1) that counteracts the induced ROS formation and changes in oxidative status [129]. An enhancing effect of 60 Hz ELF-MF (0.8 mT) on the induced immune response and NO production was found in RAW246.7 mouse macrophage tumor cells [130], whereas a reduction in NO production by LPS was reported in the same cell line exposed to 50 Hz ELF-MF (0.5 mT) [131]. These contrasting effects may be due to the different sequence of treatments. In isolated mouse macrophages, a slightly increased ROS production was observed, resembling to some extent an induced immune response, but the signaling pathways superimposed only partially [132]. This could indicate that ELF-MF does not trigger a genuine immune response, nevertheless creating a cellular situation that leads to a change in the response to further stimuli or stress situations. For example, previous exposures to 10 and 50 Hz, but not to 100 Hz, ELF-MF (1 mT) had a protective effect, reducing apoptosis and ROS formation in human microglia cells when they were metabolically stressed by deprivation of oxygen and sugar, i.e., conditions similar to those that occur in brain ischemia [133].

Evidence for an inflammatory cell response regarding iNOS activity and NO production was found in mouse microglia cells exposed to a GSM signal (SAR: 2 W/kg) [95] for 24 h or for a short time (20 min) to pulsed 2.45 GHz RF-EMF [134,135]. In both situations, activation of STAT3 (signal transducer and activator of transcription 3) and mitogen-activated protein kinase (MAPK) signaling pathways was observed, as well as changes in the production of cellular messengers and a reduction in microglial phagocytosis. It should be noted that exposure to pulsed 2.45 GHz RF-EMF with 6 W/kg SAR is a situation or signal type that would hardly occur as an environmental factor [134,135]. On the other hand, exposure of this cell type to a 900 MHz GSM signal (SAR: 4 W/kg) temporarily altered the activity of the mitochondrial cytochrome c oxidase without leading to oxidative stress [101]. A decrease in phagocytosis was also observed in RAW264.7 macrophages cells, accompanied by an RF-EMF-induced increase in NO synthesis [136]. This effect increased with the duration of exposure, regardless of whether 900 MHz, 2.45 GHz, or a combination (SAR: 80–400 mW) was applied. On the other hand, 2.45 GHz RF-EMF (SAR: 0.4 W/kg) promoted phagocytosis of co-exposed black carbon particles, changed the immune response, and increased NO production and cell toxicity [137].

In this regard, it is worth mentioning that an increase in oxidative stress by RF-EMF was observed in populations of immune cells from human blood enriched by flow cytometry [138,139,140]. However, it should be noted that, in contrast to cultured cells/cell lines, this does not necessarily reflect an immune reaction, but possibly an enhancement of the cell aging or cell death process because of a strong stress situation due to the removal from their normal environment. Furthermore, leukemic HL60 cells and differentiating CD34^+^ (surface marker “cluster of differentiation 34”) human blood stem cells (HSCs) were also investigated for effects of RF-EMF exposure on the oxidative balance. In both cell types, no evidence was found that exposure to 900 MHz GSM, 1.95 GHz UMTS, and 2.53 GHz LTE signals at SAR values of 0.5–4 W/kg led to increased ROS formation after either short (4 h) or prolonged exposure [141]. In another study with stem and other blood cells, a temporary increase in ROS formation was observed after 1 h of UMTS exposure (SAR: 40 mW/kg) [142]. This was also the case in leukemic HL60 cells, in which 900 MHz RF-EMF at a calculated SAR value of 0.25 mW/kg triggered an increase in ROS formation, which was prominently detectable after 30 min, attenuated after 4 h, and vanished after 24 h of exposure [143]. ROS levels correlated well with a temporary increase in oxidative DNA damage, as well as the energy production of the mitochondria. In the same cell line, signs of increased lipid peroxidation (MDA) were also observed when the cells were exposed to 2.45 GHz RF-EMF with 217 Hz pulses at estimated 0.1 W/kg SAR, whereas no change in GSH and GPx activity was evident [144].

### 4.3. Assessment of EMF Effects on Blood and Immune Organs

ROS play an important role in the elimination of foreign or damaged cells, while they are also involved in inflammatory reactions and the activation of the immune response [21]. Long-term inhibition and repeated activation of ROS are likely to cause health effects.

There is evidence that EMF affects the response to other (stress) factors [38,124,125,126,129,130,137]. Such a crosstalk between cell responses is important in real life, since humans and animals are exposed to different and changing stress and environmental factors, in contrast to experimental studies. For example, chemically induced oxidative stress reduced the production of ROS in animals after subsequent exposure to RF-EMF, indicating an adaptive response [38]. Observations in this direction have also been made in cell studies. For instance, it was shown that immune responses and phagocytosis were altered by RF-EMF exposure [95,134,135,136,137].

Similar to the findings reported for the central nervous system, there are indications that effects of EMF exposure are age-dependent in the lymphoid system. Very young animals could not compensate for oxidative stress, even after a recovery period, whereas this was possible in older animals after complete development of the antioxidative protective system [116]. Moreover, in cultured cells, the time of analysis of oxidative stress seems to play a role, and short-term exposure led to an increase of oxidative stress in lymphoid and leukemic cells [123,143]. This increase was mostly temporary, and the triggered processes were partly similar to a genuine immune response [132].

Overall, however, only a few animal and cell studies on the influence of EMF exposure on oxidative stress and defense of the immune system are available. At present, the data available do not allow a conclusive assessment of possible health effects. Nevertheless, dependencies on preconditions, age, and exposure duration are likely similar to the nervous system.

## 5. EMF Exposure and Oxidative Stress: Effects on Reproduction

### 5.1. In Animals

Influences of EMF on male reproductive organs and sperm, as well as their precursors, were investigated in more than 30 animal studies (Supplementary Materials, Tables S1 and S3). In Sprague Dawley rats exposed to 900 MHz RF-EMF (whole-body SAR: 0.0067 W/kg) for 1 h/day for 21 days, testicular weight decreased and various morphological changes were observed, including mitochondrial integrity, apoptosis, and increased antioxidative activity [145]. In adult Wistar rats, significant changes in sperm count and vitality, morphological changes, and increased ROS levels and lipid peroxidation in sperm and their precursor stages were found after RF-EMF exposure with a 3G/UMTS signal (SAR: 0.26 W/kg) for 45 days (2 h/day), concomitantly with a decrease in sperm with active mitochondria [146]. Similar results were reported by Shahin et al. [55]. Yet again, significant decreases in sperm count and vitality were found, which were associated with an increase in various oxidative stress markers (ROS, NO, MDA) and a decrease in antioxidative activities (SOD, GST, CAT). In addition, the amount of iNOS was increased in the precursors of sperm and in Leydig cells [55]. These findings indicate functional and morphological impairment of spermatozoa by RF-EMF exposure, associated with an increase in ROS. Liu et al. reported ROS formation and oxidative stress in rat sperm, as well as tissue changes and increased apoptosis, after exposure to 900 MHz RF-EMF (SAR: 0.66 W/kg) for 2 h/day and 50 days [147]. An increase in ROS, resulting in histological and morphological changes of testes and germ cells, as well as DNA damage, was found in Swiss mice after exposure to 900 MHz RF-EMF (SAR: 0.0054–0.0516 W/kg) twice for 3 h/day for 35 days [148].

Increased lipid peroxidation after 2.45 GHz RF-EMF exposure (50 Hz pulses, whole-body SAR: 0.14 W/kg, 2 h/day) for 3 weeks was found in testes of rats [48]. Exposure to 2.45 GHz RF-EMF (217 Hz-pulsed, whole-body SAR: 0.143 W/kg) of male Wistar rats for 30 days at 1 h/day did not change GSH levels and increased lipid peroxidation in testicular tissue, which could be counteracted by melatonin treatment [149]. Analogous findings were reported for male Wistar rats exposed to 900 MHz (pulsed, whole-body SAR: 1.2 W/kg) for 2 h/day for a total of 3 weeks. Lipid peroxidation and NO production were enhanced and GSH levels were decreased [150]. Pandey et al. reported mitochondrial damage, cellular damage, and DNA damage in spermatocytes of male Swiss mice exposed for 35 days to 900 MHz RF-EMF (SAR: 0.0045–0.0056 W/kg, attributing them to oxidative stress [151]. Exposure to a 900 MHz RF-EMF (SAR: 1.075 W/kg) for 2 h/day for 8 weeks resulted in changes in levels of MDA and the ROS scavenger GST in male Wistar rats [152]. The same authors also reported a significant increase in ROS, alterations of oxidative stress markers, DNA damage, increased apoptosis, inflammation, and tissue toxicity in testes of Swiss mice exposed to 1.8 GHz RF-EMF (SAR: 0.05 W/kg) for 120 days [153]. RF-EMF exposure of male Wistar rats at 900 MHz, 2 h/day for 35 days (SAR: 0.9 W/kg) [154], as well as 4 h/day for 20, 40, and 60 days (SAR: 0.043–0.135) [155], revealed alterations in various oxidative stress markers in the testes, with one study also demonstrating DNA damage [154]. Similar findings were obtained in male Wistar rats after exposure to a 900/1800 MHz dual-band mobile phone (no fields measured or SAR calculated) for 1, 2, or 3 h/day [156]. In one study, combined 900/1800/1900 MHz RF-EMF exposure for 15, 30, and 60 min/day for 14 days (SAR: 0.9 W/kg) resulted in changes in oxidative stress markers and tissue toxicity in testes of Wistar rats [157].

Previous insults or existing diseases, such as diabetes, can make the organism more sensitive to exogenous stressors [158]. Increased lipid peroxidation, NO production, and a decrease in GSH were found in the testicular tissue of male Wistar rats after exposure to 50 Hz ELF-MF (8.2 mT) and 2.1 GHz RF-EMF (SAR: 0.23 W/kg) for 20 min/day for 4 weeks. These effects were more pronounced in rats with diabetes than in healthy animals [158].

RF-EMF effects on female reproduction were also performed. For instance, RF-EMF exposure for 1 h/day, 5 days/week for 52 weeks at all investigated frequencies (900, 1800, 2450 MHz, SAR: 0.1 W/kg) resulted in an increase in lipid peroxidation but no significant changes in other oxidative stress markers in the uteri of female Wistar rats [112]. In the endometrium of Wistar rats, increased lipid peroxidation (MDA), NO production, and decreased measured antioxidative biomarkers (GSH, GPx, CAT) were found after exposure to a 217 Hz-pulsed 900 MHz RF-EMF, with a whole-body SAR of 0.014–4 W/kg for 30 min/day for 30 days [159]. However, the large SAR fluctuations with potential temperature increases entail some uncertainty whether the observed morphological changes, apoptosis, and immune modulation occurred due to the oxidative stress and/or by tissue warming. Similarly, increased ROS production and associated changes in oxidative stress markers were found in the uterus and the ovaries of female Swiss mice exposed to 1.8 GHz RF-EMF for 100 days. However, no SAR values were reported [51]. Another study in female mice demonstrated increased oxidative stress and morphological alterations of the implantation sites of the embryos in the placenta when female Parkes mice were exposed to 2.45 GHz RF-EMF (SAR: 0.023 W/kg) for 2 h/day for 45 days [118]. This resulted in impaired reproduction, measured as implantation failure or resorption of the embryos, which might have been caused by increased formation of ROS. This affects a very early stage of pregnancy (corresponding to days 7–8 in humans) when the blastocyst attaches to the uterine wall.

Regarding development, it is also of relevance whether exposure of dams causes oxidative stress in the fetuses and whether this results in any kind of impairments in the offspring. In the study by Özorak et al., Wistar rats were exposed to 217 Hz-pulsed 900 MHz, 1800 MHz, or 2.45 GHz RF-EMF (whole-body SAR: 0.18 W/kg; 10 V/m) for 60 min/day in the uterus and up to 6 weeks after birth [160]. For all three frequencies, lipid peroxidation in the newborns was initially decreased (week 4 after birth), while it was significantly higher at 6 weeks. Antioxidative markers in RF-EMF-exposed rats were significantly lower than those of the control animals at all three time-points measured (4, 5, and 6 weeks postpartum) and at all frequencies [160]. ROS were not analyzed, but the increased lipid peroxidation in RF-EMF exposed animals at 6 weeks of life and the decrease in antioxidative markers suggest an oxidative stress situation. Increased ROS production was also found in ovaries of female Wistar rats after 2.45 GHz RF-EMF exposure (SAR: 0.1 W/kg) for 1 h/day in utero and/or 1 h/day from postnatal day 21 to puberty [161].

### 5.2. In Cultured Cells

The functionality of cells of the reproductive system was also examined for effects of EMF (Supplementary Materials, Tables S2 and S4). Due to their temperature sensitivity, developmental characteristics, and availability, mainly male germ cells and cells from the reproductive organ were employed. Among them, two mouse cell lines, GC-1 and GC-2, representing two stages of sperm development, were used most frequently, but sperm and spermatogonia from humans and mice, as well as the testosterone-producing Leydig cells from testicular tissue, were also assessed.

The majority of studies published in the last 10 years focused on investigations of RF-EMF effects, such that hardly any recent data are available on the influence of 50 Hz ELF-MF on oxidative balance. In spermatogenic GC-1 but not in GC-2 mouse cell lines, a consistent increase in superoxide was found after exposure to 50 Hz ELF-MF (2.5 mT) for 2 h, while NO levels remained unchanged [162,163]. Here, however, changes after a recovery period of 2 days and nonimmediate responses were measured; thus, these effects are rather indications of long-term or secondary effects. The influence of prolonged ELF-MF exposure for 24 h (1, 2, 3 mT) on the genome was investigated in another study with GC-2 cells, with a marginal increase in DNA damage at the highest dose, which was interpreted as a consequence of oxidative stress [164], but data about ROS formation or oxidative stress were not provided.

Ex vivo investigation yielded ambivalent observations and conclusions in a few studies with human sperm on the influence of RF-EMF with respect to oxidative stress and quality, although similar exposure durations (45–90 min) and doses (SAR: 1–6 W/kg) were applied [165,166,167,168]. Two studies reported no signs of increased ROS, no oxidative DNA damage, or any other negative effects such as induced cell death and reduction in sperm quality, when sperm was exposed to a 900 MHz GSM or a 1.95 GHz UMTS signal [166,167]. In contrast, oxidative stress and, in some cases, massive DNA damage and loss of sperm vitality were observed after exposure to 900 MHz GSM or 2.45 GHz WiFi signal [165,168]. However, it has to be noted that the two studies without significant effects were conducted under controlled temperature and exposure conditions, while user devices were applied in the other two. It needs to be considered that exposure of cells with commercial user devices (e.g., cell phones) often entail many uncertainties, confounders, and/or fluctuations in exposure.

After 24 h of exposure with a 1.8 GHz RF-EMF (GSM signal, continuous, or intermittent), an increase in oxidative DNA damage, ROS production, and autophagy activity was observed in GC-2 cells at the highest SAR dose of 4 W/kg [164,169,170,171]. Hence, there is evidence that the increase in ROS production does not occur immediately but with increasing exposure time (>12 h) or cumulative dose [170]. Nevertheless, an increase in mitochondrial superoxide production was observed in the same cell line after 2–6 h of exposure to unmodulated 1.8 GHz RF-EMF at lower doses (SAR: 0.15 W/kg), accompanied by lipid peroxidation [172]. In this comprehensive study, these observations were confirmed in GC-1 cells and freshly isolated spermatogonia, and the origin of observed RF-EMF effects was attributed to the mitochondrial respiratory chain. Even at higher exposure intensities (SAR: 1.5 W/kg), no increase, but rather a decrease, in mitochondrial ROS formation and no change in global ROS and lipid peroxidation levels were measured in mouse spermatozoa [172]. Hence, the mouse sperm reacted differently to RF-EMF exposure than the preliminary stages of sperm development, represented by the GC-1 and GC-2 cells. The exposed sperm cells exhibited indications for oxidative DNA damage and reduced quality despite the lack of indicators of oxidative stress.

Additional evidence for an influence of RF-EMF on reproduction was obtained from studies in Leydig cells from mice, in which exposure to a 1.8 GHz GSM signal (SAR: 0.116 W/kg) or a 1.95 GHz RF-EMF (SAR: 3 W/kg) led to reduced testosterone production [173,174]. While there was evidence for oxidative stress (i.e., CAT and MDA) [173] after a short exposure for 1–3 h, an increased ROS formation was not detected after the 24 h of exposure [174]. Lastly, effects on a model system for female reproduction were investigated in cultured murine preantral follicles upon developmental induction. The exposure to 2.45 GHz RF-EMF (SAR: 0.77–0.88 W/kg) for 1 h/day negatively affected the growth and development of follicles, which was associated with increased lipid peroxidation and oxidative stress markers [175].

### 5.3. Assessment of EMF Effects on Reproduction and Fertility

The influence on fertility and the development of fetuses is an important topic, as developing organisms and cells are particularly sensitive to external stress factors. Effects of EMF on reproduction, predominantly after RF-EMF exposure, were studied in male reproductive organs and sperm and their precursor stages. In addition, dams were exposed to EMF, and possible damage in early and late stages of pregnancy, as well as in the offspring, was investigated [51,112,159].

The majority of the findings from the animal studies indicate a functional and morphological impairment of testes and spermatozoa by EMF exposure (predominantly for RF-EMF), which was associated with an increase in ROS, a reduction in the antioxidative capacity, and lipid peroxidation [48,55,146,147,148,149,150,151,153,154]. A previous insult or pre-existing disease (i.e., diabetes) was shown to be a risk factor that enhanced oxidative stress, which could not be compensated for [158]. After in utero exposure, age-dependent effects on oxidative stress markers were seen in the offspring, differing, depending on the assessed organ system [160,161]. A study on impairments at early stages of pregnancy revealed indications of reduced blastocyst implantation [118].

In cell studies, mainly male germ cells and cells from male reproductive organs were used. These are very temperature-sensitive and, therefore, temperature fluctuations must be excluded during irradiation; otherwise, false-positive findings influence the evaluation [114,115]. This was not the case in many cell studies and, therefore, it cannot be excluded that some findings are false-positive. Overall, the few cell studies do not provide any reliable evidence for an impairment of sperm cells and their precursors by EMF-induced oxidative stress, although some of them reported indications for ROS formation and oxidative stress [164,169,170,171,172].

## 6. Further Observations of Oxidative Stress Induced by EMF

In addition to the extensive literature on effects of EMF on the nervous, immune, and reproductive systems, a number of studies on oxidative stress in other organ system and cell types have been published (Supplementary Materials, Tables S1–S4).

### 6.1. Oxidative Influences on Other Organs

Evidence of adaptation to oxidative stress and antioxidant processes induced by 900 MHz RF-EMF exposure (2.5 mW/cm^2^) for 1 h/day was found in the liver and kidney of male Sprague-Dawley rats. ROS formation was increased in both organs after 60 days of RF-EMF exposure, which was associated with changes in markers of liver and kidney function. However, these changes were no longer present after 30 days of regeneration, indicating adaptation [35]. Examinations of oxidative stress in the liver of adolescent Sprague-Dawley rats after 900 MHz RF-EMF (SAR: 0.0096 W/kg) exposure for 1 h/day during postnatal days 35–59 did not result in a significant induction of ROS but led to some changes in oxidative stress markers [176]. In contrary, analyses of liver tissue at postnatal day 60 showed changes in ROS, oxidative stress markers, and tissue toxicity after in utero exposure to a 1.8 GHz (SAR: 0.12 W/kg) for 20 days and 6, 12, 24 h/day [177]. RF-EMF exposure (950 MHz) of rats, dams, and their offspring of different ages (neonates up to 30 days after birth) for up to 51 days resulted in some changes in oxidative stress and DNA damage in the liver. These effects were dependent on age, exposure duration, and dose (whole-body SARs: 0.51, 0.18, 0.18, and 0.06 W/kg for neonates, day 6, day 15, and day 30 after birth, respectively) [178]. Reduced lipid peroxidation was found only in the neonates, after RF-EMF exposure in utero, while no differences between groups were observed for protein oxidation and CAT. DNA damage was only increased in 30 day old exposed animals while it was reduced in 15 day old animals. Thus, the results on DNA damage are inconclusive and might be random due to a large variability.

Whether a pre-existing condition or disease, such as diabetes, influences the extent of oxidative stress or modulates its defense was investigated in male Sprague-Dawley rats in a diabetes model [179]. Rats with diabetes, when compared to healthy rats, showed a more pronounced production of ROS and increased lipid peroxidation in the liver after 28 days of 900 MHz RF-EMF exposure (E-field: 25 V/m) for 4 h/day. Unfortunately, no SAR value was reported in this study, and the observation of diverging activity of SOD and CAT is somewhat counterintuitive as both are markers for the antioxidative defense [179]. In the study by Esmekaya et al., lipid peroxidation and NO production were increased in the liver, as well as in the lung, heart, and kidney, of male rats after exposure to pulsed 900 MHz RF-EMF (whole-body SAR: 1.2 W/kg) for 2 h/day for 3 weeks, whereas GSH levels were decreased [150]. Shahin et al. reported changes in ROS and the oxidative stress markers SOD, CAT, and GST in the liver and kidney of pregnant Parkes mice exposed for 45 days to 2450 MHz RF-EMF (SAR: 0.023 W/kg) [118]. The same group found an increase in ROS and associated indications for oxidative stress in the liver and kidney of male Swiss mice exposed to 2450 MHz RF-EMF (SAR: 0.018 W/kg) for 30 days [55].

Increased lipid peroxidation, as well as a decrease in antioxidative markers, was observed in the kidney of rats exposed to 217 Hz-pulsed 900, 1800 MHz, or 2450 MHz RF-EMF (whole-body SAR: 0.18 W/kg; 10 V/m) for 5 days/week and 60 min/day from in utero until 6 weeks after birth [160]. Interestingly, lipid peroxidation was decreased in exposed animals at the fourth week of life, while the antioxidative biomarkers were consistently lower than those of the corresponding controls at all three time-points assessed (4, 5, and 6 weeks after birth). Applying a 2450 MHz RF-EMF, a study in Wistar rats found changes in both ROS and oxidative stress markers in the kidney [180], while no alterations were found for ROS [181]. Similarly, four studies examining oxidative stress in the kidney of Sprague-Dawley rats using 900 MHz RF-EMF signals yielded ambivalent results. Two of them reported changes in ROS formation, oxidative stress, and tissue toxicity [35,182], one demonstrated increased ROS, tissue toxicity, and apoptosis without assessing antioxidative markers [183], and one found no indication for oxidative stress although seeing kidney toxicity [184]. Other investigations in the kidney revealed changes in ROS, oxidative stress, tissue toxicity, and apoptosis, applying 2.1 GHz RF-EMF [185].

In the heart of Wistar rats, 2.45 GHz RF-EMF exposure for 5 min (50, 100, 150, 200 mW/cm^2^) or 30 days (SAR: 0.1 W/kg) resulted in changes in ROS and oxidative stress markers and increased tissue toxicity and apoptosis [186] or in more lipid peroxidation and reduced SOD, respectively [187]. Two studies in Sprague-Dawley rats examined oxidative stress in the heart applying laboratory-generated 900 MHz RF-EMF signals. After in utero exposure during gestational days 13–21 at 0.025 W/kg SAR for 1 h/day and examination at postnatal day 21, there were clear indications of oxidative stress, tissue toxicity and apoptosis in the heart [188]. In another study using young rats, increased ROS and increased apoptosis but no changes in antioxidative defense or tissue toxicity were found after 900 MHz RF-EMF exposure (SAR: 0.0093 W/kg) for 1 h/day on postnatal days 21–59 [189].

Lastly, there are some sporadic reports about oxidative stress related to RF-EMF exposure in other tissue types. For instance, 2.45 GHz WiFi exposure (whole-body SAR: 0.1 W/kg) of male Wistar rats caused increased lipid peroxidation in the mucosa of the vocal tract, while no differences in antioxidative biomarkers were measured [190]. Increased lipid peroxidation, apoptosis, and pathological tissue changes were found in the bladder of young rats exposed to 900 MHz RF-EMF (SAR: 0.0067 W/kg) [183]. Two animal studies on possible oxidative stress on the eyes have been published [119,191], both indicating no increased ROS production. Exposure to 2.45 GHz RF-EMF, pulsed at 217 Hz (whole-body SAR: 0.1 W/kg), for 1 h/day and 30 days had no marked effect on lipid peroxidation in the eye, while antioxidative biomarkers (GPx and GSH) were significantly decreased [191]. In combination with melatonin treatment, these effects were reverted, which was explained by the antioxidant effect of melatonin. In contrast, no evidence for increased oxidative stress and NO production by 1.8 GHz RF-EMF (whole-body SAR: 0.4 W/kg) was found in Wistar rats exposed 1 h/day for 3 weeks [119]. However, the exposure of animals in the cages was performed using a cell phone in talk mode, which is inevitably associated with large uncertainty and variability of the SAR.

For ELF-MF exposure, only a few animal studies with readout concerning oxidative stress have recently been published. No evidence for increased lipid peroxidation (MDA) in the liver was found by Erdal et al., in which Wistar rats of both sexes were exposed to 50 Hz ELF-MF (1 mT) for 4 h/day and 445 days [192]. The results of a study in male Wistar rats exposed to 60 Hz ELF-MF (2.4 mT) for 2 h indicated an impairment of the antioxidant defense in the heart and kidneys [193]. However, rats kept in tubes of a carrousel setup for exposure without ELF-MF showed similar levels of ROS and antioxidative markers, indicating that the stress situation caused by a containment in tubes also triggered oxidative stress. These findings demonstrate the need for appropriate sham-exposure controls for such experimentations to exclude confounding factors resulting in oxidative stress. However, this study did not indicate whether the animals were previously trained to go into the tubes to exclude this stress factor.

### 6.2. Experimental Data on the Effect of EMF on Skin, Epithelial, and Cancer Cells

Because of their function as a barrier and first line of defense against the environment, skin and epithelial cells are of interest for possible EMF effects. However, only experimental studies with cultured cells and none with animals have been performed in the last decade. A number of cell types with different functions and properties were used such as fibroblasts from the skin of rats (Rat-1), mice (NIH/3T3, McCoy), and humans (HSF) or human gingival fibroblasts. In addition, there are experimental data from human keratinocytes (NCTC-2544, HaCaT), specialized epithelial cells of the mammary gland (MCF10A), pulmonary fibroblasts from human (IMR-90, MRC-5) and hamster (V79), Chinese hamster ovary cells (CHO), cells of the human retina (RPE-1), and lens (HLE-B3) of the eye and human amniotic cells (FL, HTR-8/SV40neo).

Due to the use of a wide range of cell types and the limited number of directly comparable studies, the current picture regarding the effects of EMF exposure on skin and epithelial cells is patchy. Nevertheless, there is some evidence that EMF can lead, at least temporarily, to an increase in ROS production and oxidative stress in these cell types, whereby the majority of the data originate from cell studies in the ELF-MF range. A transient increase in ROS was observed in human keratinocytes (NCTC-2544) and in mouse embryonic fibroblasts (MEF) upon continuous exposure to 50 Hz ELF-MFs [194,195]. In keratinocytes, an increase in ROS formation was found after 1–2 h of exposure, concurrent with changes in oxidative stress markers (GSH, GPx, SOD) [194]. After 4 h of exposure, ROS measurements no longer showed any differences to control cells, but there were signs of building up the antioxidative defense. It is also remarkable that this exposure-related increase in ROS was found at low (50 and 100 µT) but not at higher field strengths, i.e., not in the range used in many other studies.

Nevertheless, increased ROS formation was also found in mouse fibroblasts after exposure to an ELF-MF (2 mT) in a time window of 2–6 h, correlating with an increase in autophagy [195]. As the exposure period progressed, the cells adapted to the exposure and no longer reacted with increased ROS production. In IMR-90 pulmonary fibroblasts exposed to strong 60 Hz ELF-MF (6 mT) for 3 days, this prolonged exposure even led to reduced ROS formation [196]. Together with fluctuations of antioxidative markers, a transient reduction of superoxide, H_2_O_2_, and NO level was reported for MRC-5 lung fibroblasts daily exposed for 1 h to strong 50 Hz ELF-MF (10 mT) for days 1–3, while a prominent increase was found after 7 days [197]. In the context of investigating the influence of 50 Hz ELF-MF on processes of wound healing and inflammation, a similar exposure duration (3–6 h) in gingival fibroblasts and in HaCaT keratinocytes showed increased iNOS expression and activity, whereas CAT activity and superoxide formation were reduced [198,199]. Similar observations were made in two other cell types, breast (MCF10A) and retinal epithelial cells (RPE-1), in which no signs or even a tendency to reduced oxidative stress were observed after ELF-MF exposure [200,201]. However, some of the measurements were performed after a longer recovery period and, therefore, presumably do not represent direct effects of exposure but rather a secondary cell response.

One research group conducted a series of studies in FL cells derived from the epithelium of the amniotic sac [202,203,204,205]. They found a slight increase in ROS in the cytoplasm and, with some delay, superoxide production in the mitochondria in the time period of 5–30 min after the beginning of exposure to a 50 Hz ELF-MF (0.4 mT) exposure [202]. In this case, however, it is likely that the exposure led to the activation of cellular signaling pathways rather than to canonical oxidative cell stress [203,204,205]. For instance, ELF-MF exposure alters the activity/excitability of epidermal growth factor (EGF) receptors in the cell membrane, thereby remodeling the MAPK pathway and subsequent cell responses.

The function of EMF-induced production of radicals as signal molecules for the activation of the MAPK signaling pathway was previously postulated for RF-EMF in a pioneering study [206]. In Rat-1 rat skin fibroblasts, a short exposure to unmodulated 875 MHz RF-EMF stimulated NADH oxidase and ROS production, thereby increasing the sensitivity of the EGF receptor and activating the MAPK pathway. Applying RF-EMF, there is further evidence of transient ROS formation and oxidative stress provided by a few studies. In murine NIH/3T3 embryonic fibroblasts, an increase in ROS was found, most pronounced after exposure to a 1.8 GHz GSM signal (SAR: 2 W/kg, 5/10 min on/off) for 1–2 h or a combination of a 837 Hz GSM and a 1.95 GHz UMTS signal (SAR: 4 W/kg) [102,207]. In contrast, the combined exposure to these two signals in MCF10A breast epithelial cells did not result in an increase in ROS and changes in oxidative stress markers [208]. Likewise, no increase of mitochondrial superoxide formation was observed for exposure to 1.8 GHz RF-EMF (SAR: 0.15 W/kg) for 2–6 h in another mouse fibroblast cell line [172]. Thus, the temporary increase in ROS does not seem to be a general cell response but specific to certain cell types.

Performed again in mouse fibroblasts, an unusually high cell mortality was found upon continuous 1.8 GHz RF-EMF exposure (1.2 W/m^2^) for 2 days [105]. In contrast to most other comparable investigations, ROS formation was not temporary and detectable shortly after the start of exposure, instead it became obvious only after 6 h and increased with exposure duration. This finding suggests that ROS formation might not be a direct result of exposure but a secondary effect, due to apoptosis. Similar mechanisms may have played a role in the ROS increase after exposure of CHO cells to a GSM-modulated 900 MHz RF-EMF (SAR 2 W/kg) for 12 and 24 h [209]. This notion is further supported by observations made in hamster (V79) and human (HSF) fibroblasts. Without having a negative effect on viability or leading to cell damage, exposure to 1.8 GHz RF-EMF (SAR: 1.6/3 W/kg, GSM signal or carrier wave) led to an early transient increase in ROS formation which ceased after 24 h of exposure [113,210,211]. In accordance with these conclusions, no evidence for oxidative DNA damage was found in pulmonary fibroblasts, regardless of the exposure duration (1, 4, 24 h) with different doses (SAR: 0.5, 2, 4.9 W/kg) and modulations of 1.95 GHz RF-EMFs (GSM, UMTS, WiFi) [212]. However, a slight reduction in cell vitality after 6–24 h of exposure to a 1.8 GHz GSM signal (SAR: 2.3 and 4 W/kg) was observed in HLE B3 lens epithelial cells, accompanied by an increase in the lipid peroxidation marker, MDA [213]. Gene expression and protein levels for key antioxidative enzymes (SOD, CAT, GPx1) were lowered. Hence, the authors concluded that the higher ROS levels measured after 30–90 min of exposure were due to a reduced activity of the antioxidant defense system, which is in contrast to other cell types, in which ROS production was attributed to stimulation of oxidizing enzymes such as the NADH oxidases by EMF.

In addition to the studies with cultured cells that could be assigned to one of the above biological functions or organs, there are also some experimental results that were generated in various primary or tumor cells of different origin. Although it is hardly possible to derive a uniform picture and comprehensive conclusions, these results provide additional information about the influence of EMF on the oxidative balance of cells. For instance, 50 Hz ELF-MF (100 µT) did not cause any change in ROS formation or GSH levels in heart muscle cells, whether after continuous or after interval exposure for a short time [214]. In contrast, in a murine squamous cell carcinoma line (AT478), exposure at 1 mT for 16 min resulted in an increase in ROS formation and in the activities of SOD and GPx, while MDA concentrations decreased [215]. However, other cancer cell lines reacted differently to 50 Hz ELF-MF exposure (6 mT) for 2 h. ROS levels remained unchanged in Gist-T1 gastrointestinal stromal tumor cells, increased in HCT-116 colorectal cancer cells, and tended to be lower in HEK293T embryonic kidney cells [201]. Continuous 60 Hz ELF-MF exposure (6 mT) of the HeLa cervical cancer cells resulted in lower ROS and better cell viability [196], whereas, in breast cancer cells, an increase in ROS formation after 2 h was found, accompanied by induced apoptosis upon prolonged exposure to the ELF-MF (1 mT) [216]. Notably, ROS formation after exposure to 200 Hz rather than 50 Hz ELF-MF was considered here, as the former generally showed stronger effects on apoptosis.

In the RF-EMF range, analogous observations were made in MDA-MB-231 breast cancer cells exposed to a 900 MHz GSM-like signal (SAR: 0.36 W/kg). RF-EMF exposure for 1 h resulted in an increase in ROS formation and induced cell death [217]. Apoptosis and more ROS was also seen in MCF-7 breast cancer cells after exposure to 217 Hz pulsed 900, 1800, and 2450 MHz RF-EMF (average SAR: 0.36 W/kg) for 1 h [111]. RF-EMF-induced cell death was also observed in embryonic kidney cells (HEK293, HEK293T) exposed to an unmodulated 940 MHz carrier wave (SAR: 90 mW/kg) [218] or 2.45 GHz (E-field: 2 V/m) [219,220] for about 1 h. However, the results from the analyses of the markers for oxidative stress differed. While the 2.45 GHz RF-EMF resulted in higher MDA levels and reduced activities of SOD and GPx, exposure to the 940 MHz RF-EMF decreased MDA values over time while increasing SOD, with maximal changes observed at 30–45 min after the exposure started. In similar time windows, a transient increase in ROS formation, accompanied by changes in oxidative stress markers, was reported after exposure of HEK293 cells to 940 MHz RF-EMF (SAR: 90 mW/kg) [218] or MC3T3-E1 osteoblastic cells to a 2.45 GHz WiFi signal (SAR: 0.16/0.85 W/kg) [221]. The reason for a reduction in cell numbers may not always be induced apoptosis but could result from the promotion of cell senescence [222]. This was observed in the population of various cancer cells, as well as in stem cells from adipose tissue, after exposure to a 1.7 GHz long-term evolution/fourth generation (LTE) signal (SAR: 1 and 2 W/kg) for 3 days. Depicted for HuH7 liver cancer cells and the adipose stem cells, the increased formation of mitochondrial and total ROS by exposure played a role in the promotion of senescence, as more cells with stronger ROS signals were present. On the other hand, exposure to 900 MHz RF-EMF (SAR: 80 or 170 mW/kg) in isolated thyroid cells neither affected cell vitality nor provided indications for oxidative stress or an increase in ROS formation [223].

In summary, EMF exposure of cultured cells does not to induce an universal cellular reaction, but a variety of mechanisms and stress responses including ROS formation and oxidative stress might be triggered, depending on cell type and experimental conditions. In this regard, it needs to be noted that established cell lines and especially cancer cells, representing the majority of the cell lines studied, might react more strongly and more variably than normal cells, likely attributed to their altered metabolism and regulatory mechanisms.

## 7. Conclusions

The majority of recent animal studies on increased ROS production and oxidative stress caused by EMF were aimed at investigations of the nervous system and reproduction. Analogously, in cell studies, neurons or neuron-like cells were most frequently used. Animal studies on oxidative stress and possible impairment of reproduction at different stages (sperm maturation, very early stages of pregnancy such as implantation, and effects in newborns and after a few weeks of EMF exposure to the mother animals during pregnancy) follow in second place. These animal studies were supported by some cell studies, mainly in mouse cell lines of the male reproductive system and in sperm. Overall, more cells than animal studies were published, using, in addition to the abovementioned cell types of the nervous and reproductive system, immune and cancer cells, as well as isolated cells from the skin and epithelia. For this report, animal and cell studies were included, according to their quality and research question, in order to give an informative overview of the available studies; however, this is not a systematic review.

In summary, indications for increased oxidative stress caused by RF-EMF and ELF-MF were reported in the majority of the animal studies and in more than half of the cell studies. Investigations in Wistar and Sprague-Dawley rats provided consistent evidence for oxidative stress occurring after RF-EMF exposure in the brain and testes and some indication of oxidative stress in the heart. Observations in Sprague-Dawley rats also seem to provide consistent evidence for oxidative stress in the liver and kidneys. In mice, oxidative stress induced by RF-EMF was predominantly demonstrated in the brain and testes, as well as in liver, kidneys, and ovaries. These observations were made with a variety of cell types, exposure times, and dosages (SAR or field strengths), within the range of the regulatory limits and recommendations. Certainly, some studies were subject to methodological uncertainties or weaknesses or are not very comprehensive regarding exposure time, dose, number, and quantitative analysis of the biomarkers used, to name a few. A trend is emerging, which becomes clear even when taking these methodological weaknesses into account, i.e., that EMF exposure, even in the low dose range, may well lead to changes in cellular oxidative balance. Organisms and cells are able to react to oxidative stress, and many observations after EMF exposure point to an adaptation after a recovery phase. Adverse conditions, such as diseases (diabetes, neurodegenerative diseases), compromise the body’s defense mechanisms, including antioxidant protection mechanisms, and individuals with such pre-existing conditions are more likely to experience health effects. The studies show that very young or old individuals can react less efficiently to oxidative stress, which of course also applies to other stressors that cause oxidative stress. Further investigations under standardized conditions are necessary to better understand and confirm these phenomena and observations.

## Supplementary Materials

The following are available online at https://www.mdpi.com/article/10.3390/ijms22073772/s1: Table S1. Animal studies with RF-EMF exposure; Table S2. Cell studies with RF-EMF exposure; Table S3. Animal studies with ELF-MF exposure; Table S4. Cell studies with ELF-MF exposure.

## Abbreviations

8-OHdG	8-Oxo-2′-deoxyguanosine, 8-oxo-G
AC	Alternating current
ATRA	All-*trans* retinoic acid
CAT	Catalase
ELF-MF	Extremely-low-frequency magnetic field
EMF	Electromagnetic field
eNOS	Endothelial nitric oxide synthase
ERK	External signal-regulated kinase
GPx	Glutathione peroxidase
GSH	Glutathione
GSM	Global system for mobile communications, 2G
GSSG	Glutathione disulfide
GST	Glutathione *S*-transferase
GR	Glutathione reductase
HSC	Hematopoietic stem cell
IARC	International Agency for Research on Cancer
iNOS	Inducible nitric oxide synthase
LPS	Lipopolysaccharide
LTE	Long-term evolution, 4G
MAPK	Mitogen-activated protein kinase
MDA	Malondialdehyde
NADPH	Nicotinamide adenine dinucleotide phosphate
nNOS	Neuronal nitric oxide synthase
NO	Nitric oxide
NOS	Nitric oxide synthase
NOX	NADPH oxidase
PMA	Phorbol-12-myristate-13-acetate
PRDx	Peroxiredoxin
RF-EMF	Radiofrequency magnetic field
ROS	Reactive oxygen species
SAR	Specific absorption rate
SOD	Superoxide dismutase
TRPV1	Transient receptor potential cation channel subfamily V member 1
UMTS	Universal mobile telecommunications system, 3G
UV	Ultraviolet
WiFi	Wireless Fidelity, WLAN standard
